# The calcilytics Calhex-231 and NPS 2143 and the calcimimetic Calindol reduce vascular reactivity via inhibition of voltage-gated Ca^2+^ channels

**DOI:** 10.1016/j.ejphar.2016.10.008

**Published:** 2016-11-15

**Authors:** Harry Z.E. Greenberg, Kazi S. Jahan, Jian Shi, W.-S. Vanessa Ho, Anthony P. Albert

**Affiliations:** Vascular Biology Research Centre, Institute of Cardiovascu lar & Cell Sciences, St. George's, University of London, Cranmer Terrace, London SW17 0RE, UK

**Keywords:** Calcium-sensing receptor, Calcilytic, Calcimimetic, Calhex-231, NPS 2143, Calindol

## Abstract

The present study investigates the effect of commonly used negative and positive allosteric modulators of the calcium-sensing receptor (CaSR) on vascular reactivity. In wire myography studies, increasing [Ca^2+^]_o_ from 1 mM to 6 mM induced concentration-dependent relaxations of methoxamine-induced pre-contracted rabbit mesenteric arteries, with 6 mM [Ca^2+^]_o_ producing almost complete relaxation. [Ca^2+^]_o_-induced relaxations were attenuated in the presence of the calcilytics Calhex-231 and NPS 2143, and abolished by the removal of the endothelium. In addition to their calcilytic effects, Calhex-231 and NPS 2143 also produced concentration-dependent inhibitions of methoxamine- or KCl-induced precontracted tone, which were unaffected by removal of the endothelium and unopposed in the presence of the calcimimetic Calindol. In vessels with depleted Ca^2+^ stores, contractions mediated by Ca^2+^ influx via voltage-gated Ca^2+^ channels (VGCCs) were inhibited by Calhex231. In freshly isolated single rabbit mesenteric artery smooth muscle cells, Calhex-231 and NPS 2143 inhibited whole-cell VGCC currents. Application of Calindol also inhibited methoxamine- and KCl-induced pre-contracted tone, and inhibited whole-cell VGCC currents. In conclusion, in addition to their CaSR-mediated actions in the vasculature, Calhex-231, NPS 2143 and Calindol reduce vascular contractility via direct inhibition of VGCCs.

## 1 Introduction

The extracellular calcium-sensing receptor (CaSR) has a well-characterised role in regulating plasma Ca^2+^ homeostasis through regulating the secretion of parathyroid hormone (PTH) from the parathyroid gland. (Brown and MacLeod, 2001, Hofer and Brown, 2003, Ward and Riccardi, 2012). The CaSR is a therapeutic target for diseases linked to calcium homeostasis, and a series of allosteric modulators of the CaSR are available (Hebert, 2006, Jensen and Bräuner-Osborne, 2007; Mancilla and De Luca, 1998; Nemeth et al., 2001; Petrel et al., 2003; Steddon and Cunningham, 2005). Positive CaSR modulators such as Cinacalcet and Calindol, termed calcimimetics, potentiate the action of extracellular Ca^2+^ at the receptor to suppress PTH release (Hebert, 2006, Jensen and Bräuner-Osborne, 2007, Steddon and Cunningham, 2005). As such, Cinacalcet (Mimpara^®^), the only allosteric modulator of Gprotein coupled receptors currently approved for clinical use, is used to treat uraemic secondary hypercalcaemia, and hyperparathyroidism, associated with parathyroid malignancy (Hebert, 2006, Jensen and Bräuner-Osborne, 2007, Steddon and Cunningham, 2005). Conversely, negative CaSR modulators such as NPS 2143 and Calhex-231, termed calcilytics, decrease stimulation of CaSRs to increase PTH release (Mancilla and De Luca, 1998; Nemeth et al., 2001; Petrel et al., 2003; Steddon and Cunningham, 2005). Calcilytics have been proposed to treat patients with gain-of-function CaSR-mutations, and osteoporosis as increases in plasma PTH levels may have anabolic effects on trabecular and compact bone (Fitzpatrick et al., 2011, Han and Wan, 2012).

Functional CaSR expression has also been demonstrated in tissues not associated with regulating calcium homeostasis, including the vasculature. Stimulation of endothelial CaSRs by increasing the external Ca^2+^ concentration ([Ca^2+^]_o_) and with calcimimetics induces nitric oxide (NO) production which leads to vasorelaxations through stimulation of BK_Ca_ channels in vascular smooth muscle cells (VSMCs) (Awumey et al., 2013, Greenberg et al., 2016, Loot et al., 2013, Smajilovic et al., 2007, Ziegelstein et al., 2006). Endothelial CaSR stimulation also activates IK_Ca_ channels to induce endothelium-derived hyperpolarisations (EDH) and vasorelaxations (Awumey et al., 2013, Dora et al., 2008, Greenberg et al., 2016, Weston et al., 2005a, Weston et al., 2008). In addition, stimulation of CaSRs expressed on perivascular neurons (Bukoski et al., 2002, Bukoski et al., 1997, Ishioka and Bukoski, 1999, Mupanomunda et al., 1998, Wang and Bukoski, 1998) and VSMCs (Li et al., 2011, Molostvov et al., 2008, Molostvov et al., 2007, Schepelmann et al., 2016, Smajilovic et al., 2006, Wonneberger et al., 2000) have also been linked to changes in vascular reactivity.

These findings indicate that calcimimetics and calcilytics may represent potential therapeutic targets to control vascular contractility. However, the mechanism of action of these agents on vascular tone is unclear, with both CaSR-dependent and -independent effects described (Bonomini et al., 2012, Loot et al., 2013, Rybczyńska et al., 2006, Rybczynska et al., 2006, Rybczynska et al., 2010, Smajilovic et al., 2007, Thakore and Ho, 2011). Calcimimetics have been shown to directly induce NO production in endothelial cells (ECs) and directly inhibit voltage-gated Ca^2+^ channels (VGCCs) in VSMCs (Bonomini et al., 2012, Thakore and Ho, 2011). Calcilytics have also been proposed to attenuate phenylephrine- and KCl-evoked contractions in mouse aorta and induce an acute hypertensive effect in vivo studies that may involve a CaSR-dependent or independent action on the vasculature (Loot et al., 2013, Rybczyńska et al., 2006, Rybczynska et al., 2006, Rybczynska et al., 2010).

Our limited understanding of the effects of CaSR modulators in the vasculature is an important omission if the CaSR is to become a potential therapeutic target to control vascular contractility. In addition, understanding how these agents affect the vasculature may predict potential adverse effects of CaSR-related drugs used to regulate PTH secretion and Ca^2+^ homeostasis. The present study addresses these issues by examining how the commonly used CaSR calcilytics, Calhex-231 and NPS 2143 affect vascular contractility at concentrations which have previously been shown to inhibit CaSR responses in a variety of tissues. The effects of the calcimimetic compound calindol on vascular reactivity are also examined. Our results reveal that in addition to regulating CaSR-mediated changes in vascular tone, these drugs also inhibit vascular reactivity via CaSR-independent mechanisms which predominantly arises from direct blockade of VGCCs. Importantly, both the CaSR-dependent and independent actions are likely to occur at similar concentrations.

## 2 Materials and methods

### 2.1 Cell and vessel segment preparation

Male New Zealand White rabbits (2.5–3 kg) were killed by intravenous injection of sodium pentobarbitone (120 mg/kg) in accordance with Schedule I of the UK Animals Scientific Procedures Act, 1986. Second-order branches of rabbit superior mesenteric artery were dissected and cleaned of adherent tissue in physiological salt solution (PSS) containing (mM): NaCl 126, KCl 6, Glucose 10, HEPES 11, MgCl_2_ 1.2, and CaCl_2_ 1.5, with pH adjusted to 7.2 with 10 M NaOH. Following dissection, vessels were either cut into 2 mm segments for wire myography studies or enzymatically dispersed to obtain freshly isolated single VSMCs. To isolate VSMCs, the vessels were cut open longitudinally and the endothelium was gently removed from the vessel wall with a cotton bud, and vessels were then washed in PSS containing 50 µm [Ca^2+^]_o_ for 5 min at 37 °C and placed in fresh 50 µm [Ca^2+^]_o_ PSS containing collagenase (1 mg/ml) and protease (0.2 mg/ml) for 15 min at 37 °C. Following this, vessels were triturated in fresh PSS and the cell-containing solution was collected and centrifuged for 1 min at 1000 rpm. The supernatant was removed and the cells re-suspended in fresh PSS containing 0.75 mM [Ca^2+^]_o_, plated onto coverslips, and left at 4 °C for 1 h before use.

### 2.2 Isometric tension recordings

The effects of increasing concentrations of [Ca^2+^]_o_, Calhex-231, NPS 2143 and Calindol on vascular tone were investigated using wire myography. Vessel segments of 2 mm in length were mounted in a wire myograph (Model 610 M; Danish Myo Technology, Aarhus, Denmark) and equilibrated for 30 min at 37 °C in 5 ml of gassed (95% O_2_/5% CO_2_) Krebs– Henseleit solution of the following composition (mM): NaCl 118, KCl 4.7, MgSO_4_ 1.2, KH_2_PO_4_ 1.2, NaHCO_3_ 25, CaCl_2_ 1, D-glucose 10. The mean resting diameter of the vessel segments was 402±6 µm (n=32 animals, 122 vessel segments, ±S.E.M). Once mounted, vessel segments were then normalized to 90% of the internal circumference predicted to occur under a transmural pressure of 100 mmHg (Mulvany and Halpern, 1977). Mean resting vessel tension following normalisation was 5.6±0.1 mN (n=32 animals, 122 vessel segments, ±S.E.M). Vessels were left for 10 min and were then challenged with 60 mM KCl for 5 min before being washed out with fresh Krebs solution. Endothelium integrity was then assessed by stably pre-contracting vessels with the α_1_ adrenoceptor agonist 10 µm methoxamine (the concentration which gives 80% of the maximal methoxamine-induced response) followed by the addition of 10 µm Carbachol (CCh). Vessels in which CCh-induced relaxations were >90% of pre-contracted tone were designated as having a functional endothelium. When necessary, endothelium was removed by rubbing the intima with a human hair, and CCh-induced relaxations of <10% of pre-contracted arteries indicated successful removal. Vessel segments were incubated for 30 min in fresh Krebs solution and then pre-contracted with 10 µm methoxamine or 60 mM KCl as required. This was followed by cumulative additions of CaCl_2_, Calhex-231 (4-Chloro-*N*-[(1 *S*,2 *S*)−2-[[(1 *R*)−1-(1naphthalenyl)ethyl]amino]cyclohexyl]-benzamide Hydrochloride), NPS 2143 (2-Chloro-6[(2 *R*)−3-[[1,1-dimethyl-2-(2-naphthalenyl)ethyl]amino-2-hydroxypropoxy]benzonitrile Hydrochloride), Calindol (N-[(1 R)−1-(1-Naphthalenyl)ethyl]−1H-indole-2-methanamine Hydrochloride; (R)−2-[[[1-(1-Naphthyl)ethyl]amino]methyl]−1H-indole Hydrochloride) or their respective vehicles. When required, inhibitors were added to the vessel segments 30 min before the construction of the concentration-response curves and were present throughout the experiments after this initial exposure period.

Experiments carried out in the presence of an inhibitor following an initial 30 min exposure period are described throughout as performed ‘in the presence of’ the relevant inhibitor tested.

In a separate set of experiments, the inhibitory effects of Calhex-231 on vascular contractility mediated by Ca^2+^ influx through activation of VGCCs was examined in endothelium removed vessels following depletion of intracellular Ca^2+^ stores. Vessels were equilibrated in Krebs solution containing 0 mM [Ca^2+^]_o_, and the cell-permeable Ca^2+^ chelator BAPTA-AM (50 µm) was added to the bath followed by a series of 10 µm methoxamine additions until no contractile response was observed. Vessels were then washed in fresh Ca^2+^-free Krebs solution together with 3 µm or 10 µm Calhex-231, or its vehicle dimethyl sulfoxide (DMSO) and left for 30 mins. This was followed by the addition of 10 µm methoxamine, and after 3 mins, 2 mM [Ca^2+^]_o_ was added to the bath.

For each experiment described above, controls were performed using vessel segments isolated from the same animal. All relaxant responses are expressed as percentage relaxation of pre-contracted tone induced by either 10 µm methoxamine or 60 mM KCl. In the Ca^2+^ influx experiment, contractions are expressed as a percentage of the maximum contraction induced by 2 mM [Ca^2+^]_o_ following stimulation with 10 µm methoxamine, in the absence of any drug or vehicle (DMSO). Control vessels also had their calcium stores depleted and were lacking a functional endothelium. Data points on all graphs and bars on all bar charts are mean values and error bars represent S.E.M. For each experiment *n*=number of animals, with at least 3–4 vessel segments used from each animal.

Responses were analysed by 2-Way ANOVA followed by Bonferroni *post hoc* tests or by Student's *t-*test where appropriate. Where required, drug concentrations inhibiting 50% of the contraction (IC_50_) as well as the maximal inhibition (E_max_) were calculated from an inspection of the dose-response curves and compared using Student's *t*-test. In all analyses, P<0.05 was taken as statistically significant. Bonferroni comparisons are shown above the graph data points whereby: *P<0.05, **P<0.01, ***P<0.001, ****P<0.0001 vs. controls. Statistical analyses and graphs were made using Graphpad Prism 6 software (GraphPad Software, Inc, San Diego, CA, USA).

### 2.3 Electrophysiology

The whole-cell configuration of the patch clamp technique was used to record VGCC currents. In voltage–clamp mode, currents were evoked by a single depolarising step from a holding potential of −70 mV to +20 mV for a duration of 350 ms. In these experiments barium was used as the charge carrier, as barium is more permeant than Ca^2+^ through VGCCs and reduces Ca^2+^-induced inactivation. The external bath solution contained (mM): BaCl_2_ 110, HEPES 10, Glucose 11, TEA 10 (pH adjusted to 7.4 with Ba(OH)_2_). The pipette solution contained (mM): Cesium glutamate 140, EGTA 10, MgCl_2_ 2.5, MgATP 2.5, with pH adjusted to 7.4 with CsOH. Recordings were made with an Axopatch 200B amplifier (Axon Instruments, Union City, CA, USA) at room temperature (20–23 °C). Currents were filtered at 1 kHz (−3 dB, low-pass 8-pole Bessel filter, Frequency Devices model LP02; Scensys, Aylesbury, UK) and sampled at 5 kHz (Digidata 1322 A and pCLAMP 9.0 software; Molecular Devices, Sunnydale, CA, USA). Data from n=7 patches and at least 3 animals were analysed using paired Student's *t*-test with P<0.05 considered significant. Figures and analyses were made using MicroCal Origin 6.0 software (MicroCal Software, Northampton, MA, USA).

### 2.4 Materials

All materials were purchased from Sigma-Aldrich (Sigma Chemical Co., Poole, UK) or Tocris (Tocris Biosciences, Bristol, UK). Drugs were dissolved in distilled water or DMSO.

## 3 Results

### 3.1 Calhex-231 and NPS 2143 inhibit both CaSR-mediated vasorelaxations and peak amplitudes of methoxamine pre-contracted rabbit mesenteric arteries

**Fig. 1:**
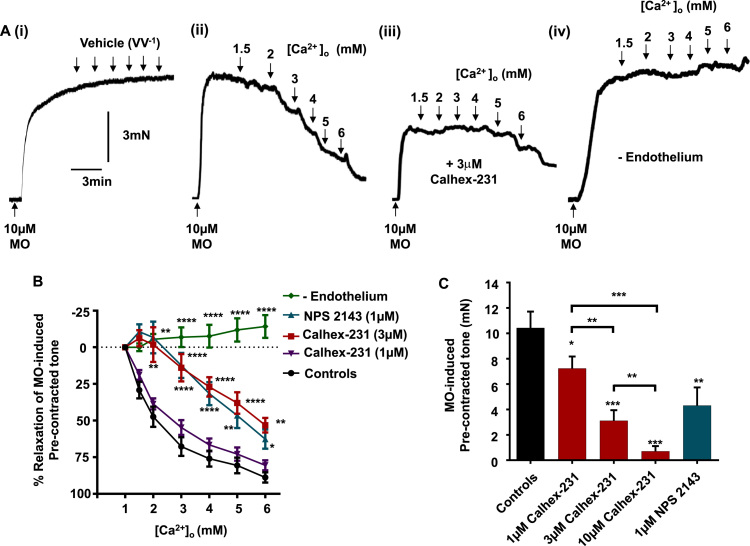
Effect of CaSR stimulation and calcilytics on pre-contracted rabbit mesenteric arteries. (*A*) Representative traces showing the effect of increasing [Ca^2+^]_o_ on pre-contracted arteries induced by 10 µm methoxamine (MO). Record (i) shows that MO induces a stable vasoconstriction in the presence of 1 mM [Ca^2+^]_o_, and the effect of adding increasing volumes of distilled H_2_O to the myograph bath. Record (ii) shows the effect of increasing [Ca^2+^]_o_ from 1 mM to 6 mM. Record (iii) shows that the presence of Calhex-231, inhibits both [Ca^2+^]_o_-induced vasorelaxations and amplitude of methoxamine-induced vasoconstriction. Record (iv) demonstrates the effect of removing the endothelium on Ca^2+^]_o_-induced vasorelaxations. (*B*) Mean data showing that [Ca^2+^]_o_-induced vasorelaxations are attenuated in the presence of 3 µm Calhex-231, 1 µm NPS 2143, and removal of the endothelium. 1 µm Calhex-231 however is without effect. **P<0.01, ***P<0.001, ****P<0.0001, 2-way ANOVA followed by Bonferroni *post hoc* test. *(C)* Mean data showing the effect of 1, 3 and 10 µm Calhex-231 or 1 µm NPS 2143 on mean amplitude of the methoxamine-induced pre-contracted arteries. Each point is from n=4 animals, with at least n=3 vessel segments per animal. Student's *t-*test, **P<0.01.

We initially performed wire myography studies to confirm that stimulation of endothelium CaSRs induced vasorelaxations of pre-contracted rabbit mesenteric arteries (Greenberg et al., 2016). Fig. 1*A*(i), (ii) and *B* show that increasing [Ca^2+^]_o_ from 1 mM to 6 mM induced concentration-dependent vasorelaxations of methoxamine pre-contracted arteries compared to vehicle controls, with 6 mM [Ca^2+^]_o_ inducing almost complete relaxation. To confirm that [Ca^2+^]_o_-induced vasorelaxations were mediated by stimulation of CaSRs, vessel segments were pre-treated with the calcilytics Calhex-231 and NPS 2143 at concentrations commonly used to study CaSR responses (Faure et al., 2009, Johansson et al., 2013, Nemeth et al., 2001, Petrel et al., 2004, Petrel et al., 2003, Saidak et al., 2009, Yamamura et al., 2015, Yamamura et al., 2013, Yamamura et al., 2012). Fig. 1*A*(iii) *and B* show that [Ca^2+^]_o_-induced relaxations were significantly attenuated in the presence of 3 µm Calhex-231 and 1 µm NPS 2143, whereas 1 µm Calhex231 had no effect. Moreover, Fig. 1*A*(iv) and *B* also demonstrate that removal of the endothelium abolished [Ca^2+^]_o_-induced vasorelaxations. These findings confirm that stimulation of CaSRs induces endothelium-dependent vasorelaxations of pre-contracted arteries as previously described (Awumey et al., 2013, Greenberg et al., 2016; Weston et al., 2005, 2008).

Intriguingly, Fig. 1*A*(iii) and *C* also identify that the peak amplitude of the methoxamine induced vasoconstrictions was significantly attenuated in the presence of 1 µm, 3 µm, and 10 µm Calhex-231 in a concentration-dependent manner, and by 1 µm NPS 2143. This observation suggests that either CaSRs are involved in augmenting methoxamine-induced vasoconstrictions or that these calcilytics inhibit vascular reactivity via CaSR-independent mechanisms. Importantly, these results indicate that the negative effects of these calcilytics on vascular reactivity occur at similar concentrations to those required to reduce [Ca^2+^]_o_ induced relaxations.

### 3.2 Calhex-231 evokes vasorelaxations of methoxamine- and KCl-induced precontracted tone via CaSR-independent mechanisms

To investigate the potential CaSR-dependent and -independent effects of calcilytics on vascular contractility, we studied the action of Calhex-231 on methoxamine- and KCl- precontracted arteries. Methoxamine- and KCl-induced contractions are greatly reduced by VGCC blockers indicating that these contractions are primarily mediated by Ca^2+^ influx via activation of VGCCs (Supplementary Fig. 1). 60 mM KCl also clamps the membrane potential of vessels at about −20 mV which prevents hyperpolarisation-mediated mechanisms that are associated with CaSR-mediated vasorelaxations (Greenberg et al., 2016; Weston et al., 2005, 2008).

**Fig. 2:**
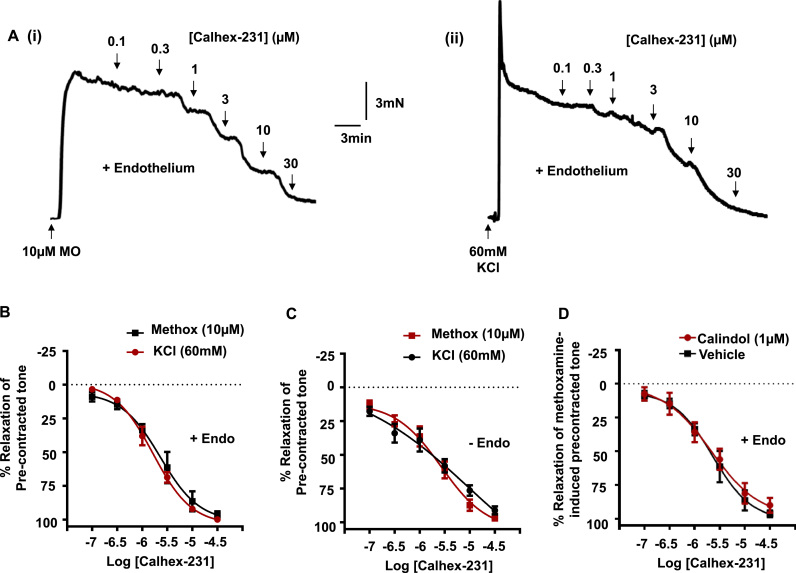
Effect of Calhex-231 on pre-contracted arteries. (*A*) Representative traces showing the effect of increasing concentrations of Calhex-231 on pre-contracted tone induced by 10 µm methoxamine (i) or 60 mM KCl (ii). Mean data showing the effect of Calhex-231 on precontracted endothelium-intact (*B*) and endothelium-removed (*C*) rabbit mesenteric arteries. Removal of the endothelium did not attenuate the Calhex-231-induced responses on either methoxamine- or KCl-induced pre-contracted arteries. (*D*) Mean data in endothelium-intact vessels showing that the presence of 1 µm Calindol also did not affect Calhex-231-induced responses on methoxamine-induced pre-contracted arteries. Each point is from n=4 animals, with at least n=3 vessel segments per animal.

In vessels containing a functional endothelium, Fig. 2*A*(i), (ii), *B* and Table 1 show that Calhex-231 evoked concentration-dependent inhibitions of both methoxamine- and KCl induced pre-contracted tone, with similar IC_50_ values and E_max_ values about 2 µm and 98% respectively. To establish whether endothelial CaSRs were involved in mediating the inhibitory responses to Calhex-231, we repeated these experiments in vessels lacking a functional endothelium. Fig. 2*C and* Table 1 show that removing the endothelium had no effect on IC_50_ or E_max_ values of the inhibitory effects of Calhex-231 on methoxamine- or KCl induced pre-contracted tone. In vehicle control experiments, DMSO had no effect on either methoxamine or KCl-evoked pre-contracted tone (*data not shown*). We also hypothesised that if CaSRs were involved in mediating inhibitions of pre-contracted tone by Calhex-231 then these effects may be opposed by the calcimimetic Calindol. Fig. 2*D* and Table 1 show that pre-treating vessels with Calindol, at a concentration known to stimulate CaSRs (Faure et al., 2009, Petrel et al., 2004, Thakore and Ho, 2011) did not affect the inhibitory effect of Calhex-231 on methoxamine-induced pre-contracted tone.

Taken together, these findings therefore indicate that the inhibitory action of Calhex-231 on methoxamine- or KCl- pre-contracted arteries are unlikely to be mediated by CaSRs.

### 3.3 Calhex-231 inhibits extracellular Ca^2+^-influx via VGCCs in store-depleted vessels

**Fig. 3:**
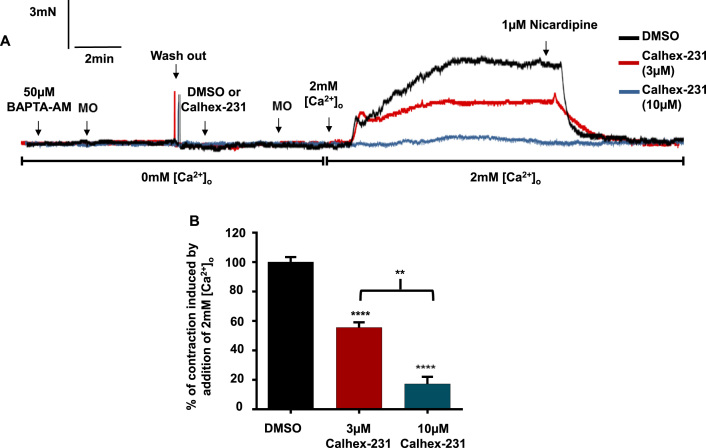
Effect of Calhex-231 on contractions induced by Ca^2+^ influx via activation of VGCCs. (*A*) Representative trace and (*B*) mean data showing that in endothelium-removed vessels with depleted Ca^2+^ stores, Calhex-231, but not it's vehicle DMSO, inhibits contractions induced by the addition of 2 mM [Ca^2+^]_o_ following stimulation with methoxamine (see text for experimental detail). n=4 animals, with at least n=3 vessel segments per animal, Student’s *t*-test, **P<0.01, ****P<0.0001. Contractions are expressed as % of the maximum contraction induced by the addition of 2 mM [Ca^2+^]_o_ following stimulation with methoxamine in endothelium-denuded control vessels with depleted Ca^2+^ stores, in the absence of Calhex-231 or DMSO.

Since both methoxamine- and KCl-evoked vasoconstrictions are predominantly dependent on activation of VGCCs (Supplementary Fig. 1), we thought that the CaSR-independent reduction of pre-contracted tone by Calhex-231 may be via inhibition of these channels. To investigate this idea further, we examined the effect of Calhex-231 on vascular contractility using a protocol in which agonist-evoked vascular tone is driven by Ca^2+^ influx through activation of VGCCs, and is unlikely to involve release of Ca^2+^ from internal Ca^2+^ stores (Thakore and Ho, 2011). Fig. 3*A* shows that vessel segments lacking a functional endothelium were equilibrated in 0 mM [Ca^2+^]_o_ Krebs solution to prevent Ca^2+^ influx, and then BAPTA-AM was applied to deplete Ca^2+^ stores. In these conditions, methoxamine did not induce contractile responses, indicating that Ca^2+^ stores had been successfully depleted. Fig. 3*A* and *B* show that following stimulation with methoxamine, increasing [Ca^2+^]_o_ to 2 mM induced stable vasoconstrictions that were inhibited by the VGCC blocker nicardipine (~100% inhibition) and by the presence of 3 µm and 10 µm Calhex-231 by around 40% and 80% respectively. In vehicle control experiments, the presence of DMSO did not affect the vasoconstrictions induced by 2 mM [Ca^2+^]_o_. These findings provide evidence that Calhex-231 inhibits agonist-induced vasoconstrictions via inhibition of VGCCs.

### 3.4 Calhex-231 inhibits VGCCs

**Fig. 4:**
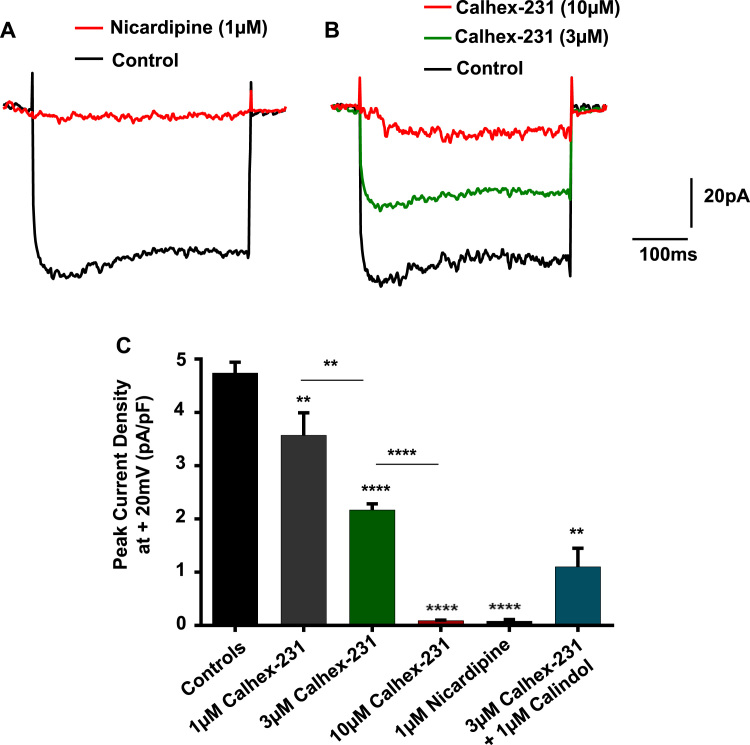
Effect of Calhex-231 on VGCC activity. (*A*) Representative trace showing that the VGCC blocker nicardipine inhibited whole-cell inward currents activated by a single depolarising step from a holding potential of −70 mV to +20 mV for a duration of 350msecs in a freshly isolated single rabbit mesenteric artery VSMC. (*B*) Representative trace and (*C*) bar graph of mean data showing that Calhex-231 inhibited whole-cell VGCC currents in a concentration-dependent manner. Inhibitions of VGCC currents by 3 µm Calhex-231 were unopposed by Calindol. For each experiment, currents were recorded from 7 patches from n=3–4 animals. **P<0.01, ****P<0.0001, Student's *t*-test.

To provide more direct evidence that Calhex-231 inhibits VGCCs, we investigated the effect of this calcilytic on VGCC currents from freshly isolated single rabbit mesenteric artery VSMCs using whole-cell patch clamp recordings. Fig. 4*A* and *C* show that application of depolarising steps from a holding potential of −70 mV to +20 mV for a duration of 350msec elicited whole-cell inward currents, which reached peak amplitude after about 10-20msec before decaying slightly to a sustained level and were inhibited by nicardipine. There was no indication of background currents being sensitive to nicardipine at the holding potential which suggests that these whole-cell VGCC currents were activated by the applied voltage steps. In time-control experiments, there was no significant decrease in the peak amplitude of inward currents following repeated depolarising steps every 20 s for 2 min (*data not shown*). Fig. 4 shows that bath application of 1, 3 and 10 µm Calhex-231 significantly inhibited mean VGCC current densities by about 25%, 60% and 95% respectively. Fig. 4*C* also reveals that inhibitions of VGCC currents by 3 µm Calhex-231 were unaffected by the presence of 1 µm Calindol.

### 3.5 NPS 2143 also induces vasorelaxations by inhibiting VGCCs

**Fig. 5:**
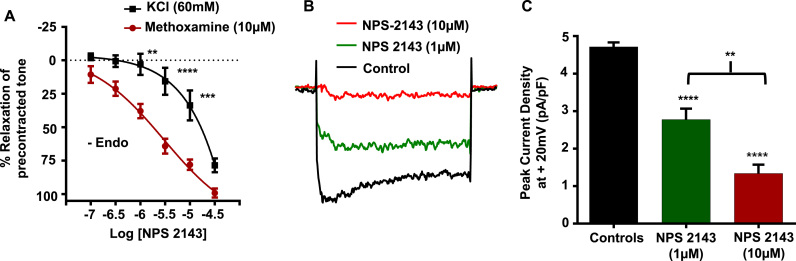
Effect of NPS 2143 on pre-contracted tone and VGCC activity. (*A*) Mean data showing that NPS 2143 induced vasorelaxations of methoxamine-induced pre-contracted endothelium removed vessels. Note that NPS 2143-induced vasorelaxations of KCl-induced contractions are attenuated compared to the effects on methoxamine-induced contractions. n=4 animals used per experiment, n=3 vessel segments per animal. **P<0.01, ***P<0.001, ****P<0.0001, 2-way ANOVA followed by Bonferroni post hoc test. (*B*) Representative trace and *(C)* mean data showing that increasing concentrations of NPS 2143 inhibited whole-cell VGCC currents. n=7 patches from n=4 animals. **P<0.01, ****P<0.0001, Student's *t*-test.

In our next series of experiments, we investigated whether the inhibitory action of NPS 2143 on agonist-evoked contractions observed earlier (see Fig. 1) also resulted from inhibition of VGCCs. Fig. 5*A* and Table 1 show that NPS 2143 induced a concentration-dependent inhibition of methoxamine-induced pre-contracted tone lacking a functional endothelium, with IC_50_ and E_max_ values of about 3 µm and 99% respectively. Fig. 5*A* and Table 1 also show that NPS 2143 inhibited KCl-induced pre-contracted tone. However, NPS 2143 was significantly less potent at inhibiting KCl-induced pre-contracted tone (IC_50_ and E_max_ values of about 13 µm and 80% respectively) compared to methoxamine-induced precontracted tone. Finally, Fig. 5*B* and *C* show that 1 µm and 10 µm NPS 2143 inhibited whole-cell VGCC currents by about 30% and 75% respectively.

Taken together, these findings strongly indicate that, similar to Calhex-231, NPS2143 also reduces vascular tone by directly inhibiting VGCCs. Importantly the inhibition of vascular reactivity by Calhex-231 and NPS 2143 occurs at concentrations previously shown to reduce CaSR-mediated responses in other cell and tissue types, with IC_50_ values of ~0.4 µm reported for both of these compounds, and maximum inhibition of CaSR responses achieved between ~3–10 µm (Faure et al., 2009, Jensen and Bräuner-Osborne, 2007, Johansson et al., 2013, Nemeth et al., 2001, Petrel et al., 2004, Petrel et al., 2003, Saidak et al., 2009, Yamamura et al., 2015).

### 3.6 Calindol induces vasorelaxations by inhibiting VGCCs

**Fig. 6:**
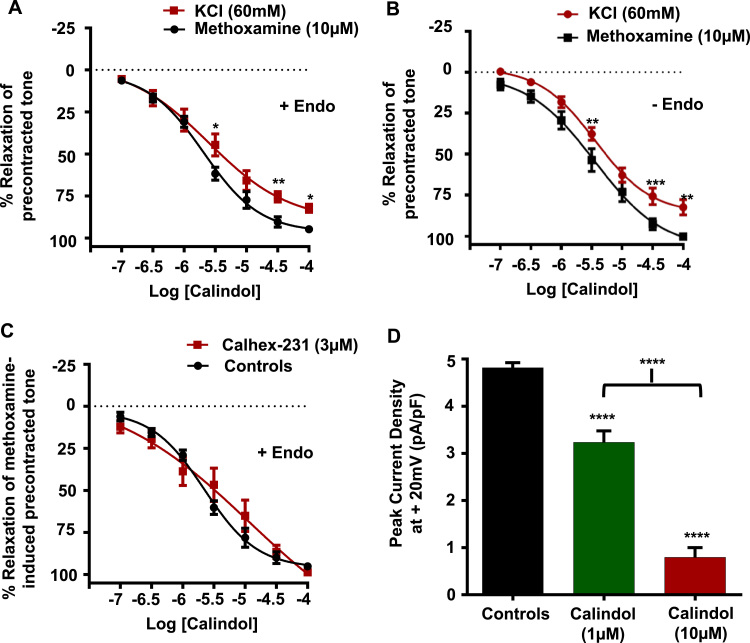
Effect of Calindol on pre-contracted arteries and VGCC activity. (*A*) and (*B*) Mean data showing that Calindol induced vasorelaxations of methoxamine-induced pre-contracted endothelium-intact and endothelium-removed vessels. Note that Calindol-induced vasorelaxations are slightly attenuated when vessels are pre-contracted with 60 mM KCl. (*C*) Mean data showing that the presence of 3 µm Calhex has no effect on Calindol-induced relaxations of methoxamine-evoked arteries. **P<0.01, ***P<0.001, ***P<0.001, 2-way ANOVA followed by Bonferroni *post hoc* test. (*D*) Mean data showing that increasing concentrations of Calindol inhibited whole-cell VGCC currents. n=7 patches from n=3–4 animals. ****P<0.0001.

In our final experiments, we investigated the vascular actions of the calcimimetic Calindol on methoxamine- and KCl-induced pre-contracted arteries, and whole-cell VGCC currents. Fig. 6*A*, B and Table 1 show that Calindol evoked concentration-dependent inhibitions of methoxamine- and KCl-induced pre-contracted tone which were unaffected by removal of the endothelium. In addition, Fig. 6*C* shows that the presence of 3 µm Calhex-231 had little effect on these inhibitory actions of Calindol. Moreover, Fig. 6*D* shows that 1 µm and

10 µm Calindol significantly inhibited mean VGCC current densities by about 30% and 80%. These findings provide strong evidence that, similar to the calcilytics Calhex-231 and NPS 2143, Calindol inhibits pre-contracted arteries independently of the CaSR by directly inhibiting VGCCs at concentrations commonly used to stimulate CaSR-mediated responses (EC_50_ ~1 µm) (Jensen and Bräuner-Osborne, 2007, Nemeth et al., 1998, Petrel et al., 2004, Saidak et al., 2009, Thakore and Ho, 2011).

## 4 Discussion

The present study demonstrates that the calcilytics Calhex-231 and NPS 2143, regulate vascular tone in rabbit mesenteric arteries via two distinct pathways; they modulate CaSRs involved in producing endothelium-dependent vasorelaxations, and inhibit agonist-evoked contractions by directly blocking VGCCs. These CaSR-independent effects of calcilytics on vascular reactivity are novel findings, which may have important implications for the use of these CaSR modulators in treating disease. In addition, the present study demonstrates that the calcimimetic Calindol also inhibits VGCCs, providing further insight into the known CaSR-independent actions of this compound.

### 4.1 Calhex-231 and NPS 2143 regulate pre-contracted arteries via CaSR-dependent and -independent pathways

In the vasculature, CaSRs are expressed in ECs, VSMCs, and perivascular neurones, and stimulation of these receptors has been linked to vasoconstrictions and vasorelaxations (Bukoski et al., 1997, Greenberg et al., 2016, Schepelmann et al., 2016; Weston et al., 2005; Ziegelstein et al., 2006). In the present study, increasing [Ca^2+^]_o_ from 1 mM to 6 mM evoked dose-dependent vasorelaxations of pre-contracted rabbit mesenteric arteries which were significantly inhibited by Calhex-231 and NPS 2143, and abolished by the removal of the endothelium. These findings are in agreement with several studies showing that stimulation of endothelial CaSRs induces NO- and EDH-mediated vasorelaxations of precontracted arteries (Awumey et al., 2013, Dora et al., 2008, Greenberg et al., 2016; Weston et al., 2005, 2008).

The current work revealed the surprise findings that applications of the calcilytics Calhex231 and NPS 2143 markedly inhibited methoxamine- and KCl-induced pre-contracted tone. In investigating these responses further, we determined four points of evidence which indicate that Calhex-231 and NPS 2143 regulate vascular reactivity via CaSR-independent mechanisms. First, the present work shows that stimulation of CaSRs evokes vasorelaxations of methoxamine pre-contracted arteries, and therefore calcilytics acting at CaSRs would not be expected to produce similar actions. Secondly, previous data showed that stimulation of CaSRs by increasing [Ca^2+^]_o_ did not affect KCl-induced contractions, implying that CaSR-mediated vasorelaxations are produced by hyperpolarisations, which are prevented by 60 mM external KCl that clamps the membrane potential of vessels at about −20 mV. However, we show that KCl-induced contractions were inhibited by Calhex-231 and NPS 2143, which suggests that these calcilytics are likely acting via different mechanisms to those produced by stimulation of CaSRs. Thirdly, we show that removing the endothelium abolishes the influence of endothelial CaSRs on vascular tone, but Calhex231 and NPS 2143 reduced pre-contracted tone in the absence of a functional endothelium. Finally, the calcimimetic Calindol may be expected to oppose the inhibitory actions of Calhex-231 on vascular tone if this effect was mediated by CaSRs. However, reduction of vascular tone by Calhex-231 was maintained in the presence of Calindol.

Taken together, these results support the conclusion that Calhex-231 and NPS 2143 have an inhibitory effect on vascular contractility via a CaSR-independent mechanism.

### 4.2 CaSR-independent actions of Calhex-231, NPS 2143, and Calindol on precontracted arteries are through inhibition of VGCCs

Methoxamine- and KCl-evoked pre-contracted tone measured using wire myography are almost completely inhibited by VGCC blockers, and therefore these contractions are likely to be predominantly mediated by Ca^2+^ influx produced by activation of VGCCs. We therefore hypothesised that the CaSR-independent inhibitory actions of Calhex-231 and NPS 2143 on vascular tone may result from direct inhibition of VGCCs. In support of this hypothesis, we observed that in vessels with depleted Ca^2+^ stores, Calhex-231 significantly impaired [Ca^2+^]_o_-induced vasoconstrictions mediated via Ca^2+^ influx through activation of VGCCs. Moreover, Calhex-231 inhibited whole-cell VGCC currents in freshly isolated single rabbit mesenteric artery VSMCs, an effect that was unopposed by the presence of calindol and NPS 2143 likewise inhibited whole-cell VGCC currents, providing the most direct evidence so far that these calcilytics act as VGCC blockers.

Interestingly, NPS 2143 was significantly more potent at inhibiting methoxamine-induced contractions compared to KCl-evoked responses. The precise reason for this is unclear, but may suggest that part of the NPS 2143 inhibition of methoxamine-induced contractions involves VSMC membrane hyperpolarisations.

The present study also provides similar evidence for the calcimimetic Calindol modulating vascular tone through a CaSR-independent inhibition of VGCCs. Calindol inhibited methoxamine- and KCl-induced pre-contracted tone in a concentration-dependent manner, which was insensitive to removal of the endothelium. The actions of Calindol on vascular reactivity were also unaffected by the presence of Calhex-231, which would be expected to oppose the actions of Calindol if they were mediated via the CaSR. Moreover, Calindol inhibited whole-cell VGCC currents in VSMCs. Together, these findings support earlier findings proposing that Calindol inhibits VGCCs to reduce agonist-evoked contractions (Thakore and Ho, 2011).

### 4.3 Significance of results in relation to functional studies

Our results indicate that the CaSR-independent effects of Calhex-231, NPS 2143, and Calindol occur at concentrations considered appropriate for studying CaSR responses (Nemeth et al., 2001, Nemeth et al., 1998, Petrel et al., 2004, Petrel et al., 2003). Importantly, CaSR-independent reduction of pre-contracted tone and VGCC currents by Calhex-231 also occurred at 1 µm, a concentration that had no effect on CaSR-mediated vasorelaxations (Fig. 1, Fig. 4). Moreover, at 300 nM Calhex-231, significant calindol-insensitive impairment of both KCl and methoxamine contractions was also observed (Fig. 2). Taken together, this suggests that there is unlikely to be differing concentration windows for the CaSR-dependent and independent actions of the agents used in this study. This conclusion has important implications when evaluating functional studies investigating the role of CaSRs in the vasculature, and also in other cells which express VGCCs.

In previous studies, inhibitory effects of calcilytics on agonist-evoked pre-contracted tone have been interpreted as CaSRs having an important role in augmenting these responses (Loot et al., 2013, Yamamura et al., 2016). Likewise, calhex-231 was shown to inhibit oxytocin-induced contractions of rat uterine tissue, with a role for the CaSR in augmenting these contractions therefore suggested (Crankshaw et al., 2013). Our findings suggest that these observations may also reflect an off-target inhibitory action of calcilytics at VGCCs. Nevertheless, vascular smooth muscle specific CaSR KO mice do exhibit a small but significant reduction in phenylephrine- and KCl-evoked vasoconstrictions and blood pressure (Schepelmann et al., 2016). It will be interesting to investigate if calcilytics produce further reductions in agonist-evoked contractions and blood pressure in these CaSR KO mice.

Intriguingly, treatment with NPS 2143 has also been shown to induce an acute increase in arterial pressure of normotensive rats (Rybczyńska et al., 2006, Rybczynska et al., 2006, Rybczynska et al., 2010) which is in contrast to the effects predicted from our findings. However, this hypertensive effect was only observed in the presence of parathyroid glands, which may suggest that the effect of NPS 2143 was due to changes in PTH levels following CaSR inhibition rather than a direct vascular action. Additionally, negative modulation of the CaSR has also been proposed to increase levels of renin, which could account for the acute hypertensive phenomenon following NPS 2143 administration (Atchison et al., 2010, Smajilovic et al., 2011).

Our findings may also signify potential cardiovascular side effects when using calcilytics in treating diseases such as osteoporosis and hypocalcaemia from gain-of-function CaSR mutations (Hebert, 2006, Jensen and Bräuner-Osborne, 2007, Saidak et al., 2009). In addition, calcilytics have been proposed as a treatment strategy for idiopathic pulmonary arterial hypertension (IPAH), where CaSR expression is proposed to up-regulated and involved in proliferation of VSMCs and increased arterial tone (Li et al., 2011, Yamamura et al., 2016, Yamamura et al., 2015, Yamamura et al., 2013, Yamamura et al., 2012). Our data suggest that these observations might result from inhibition of VGCCs by calcilytics rather than through CaSR inhibition. In agreement, several VGCC antagonists have been shown to inhibit proliferation of VSMCs in IPAH (Rich et al., 1992, Sitbon et al., 2005). Recently it has also been suggested that calcilytics could be repurposed for treating asthma, as upregulated CaSR-signalling has been shown to mediate increased airway hyperresponsiveness in an asthmatic mouse model and in human lung tissue isolated from asthmatic patients (Yarova et al., 2015). Potential cardiovascular side effects when using calcilytics may therefore also be relevant for this suggestion, and nonspecific actions of calcilytics on VGCCs may also partly explain why nebulised calcilytics blunt bronchoconstriction in the animal model of asthma tested in the study.

Calcimimetic compounds have emerged as valuable therapeutic agents in the treatment of hyperparathyroidism caused by loss-of-function CaSR mutations as well as in parathyroid cancer (Hebert, 2006, Jensen and Bräuner-Osborne, 2007, Nemeth et al., 1998, Saidak et al., 2009). Similar to our findings with calcilytics, we propose that caution should be considered when interpreting functional studies using calcimimetics. Accordingly, it is possible that the chronic hypotensive effect that follows treatment with calcimimetics in rat models of renal failure could result from the inhibitory action of these compounds on VGCCs (Carrasco et al., 2004, Ogata et al., 2003, Odenwald et al., 2006; Rybczynska et al., 2005, Rybczyńska et al., 2006, Rybczynska et al., 2006). This action may also explain the observation that calcimimetics protect against arterial calcification (Alam et al., 2009), as this affect has also been observed in studies using classical VGCC inhibitors (Chen et al., 2010).

Interestingly, this study reveals that both calycilytic and calcimimetic modulators of CaSRs reduced pre-contracted tone by inhibiting VGCCs. It is likely that molecular similarities account for this, as NPS 2143, Calhex-231, and Calindol are structurally-related to the phenylalkylamines, sharing a positively charged amino group (Jensen and Bräuner Osborne, 2007; Nemeth et al., 2001, Nemeth et al., 1998; Petrel et al., 2003; Saidak et al., 2009). Indeed, the lead compound used in the development of calcimimetics such as NPS R-568, Cinacalcet, and Calindol, was the phenylalkylamine Fenidiline, which is a potent VGCC blocker that displays weak allosteric potentiation of the CaSR (Jensen and Bräuner Osborne, 2007; Nemeth et al., 2001, Nemeth et al., 1998; Petrel et al., 2003; Saidak et al., 2009). Further screening of a series of compounds based on the phenylalkylamine structure led to the discovery of NPS 2143, and the subsequent development of Calhex-231 (Jensen and Bräuner-Osborne, 2007, Nemeth et al., 2001, Petrel et al., 2003). This structural similarity is highlighted by the fact that NPS 2143, Calhex-231, NPS R-568, Cinacalcet, and Calindol all target a common allosteric site within the seventh transmembrane domain of the CaSR. Calcilytics based on an unrelated quinazolinone structure have also been developed, namely ATF936 and AXT914 (John et al., 2014, Letz et al., 2014). In the future, it will be interesting to investigate if these agents also have CaSR-dependent and -independent actions in the vasculature, and whether they inhibit VGCC channels.

## Figures and Tables

**Table 1:** The effect of the various modulators tested on pre-contracted rabbit mesenteric arteries. Data are presented as mean values±SEM. IC_50_ and E_max_ values were compared with using Student's *t-*test. *n*=4 animals used for each experiment, with at least 3 vessel segments per animal.

**Calhex-231 on 10** **µM Methoxamine pre-contracted tone**				

	**IC**_**50**_**(µM)**	**E**_**max**_**(%)**		
**+Endothelium**	2.01±0.61	96.2±2.3		
**−Endothelium**	2.00±0.27	94.4±3.1		
**+1** **µM Calindol**	2.08±0.19	92±5.4		

**Calhex−231 on 60** **mM KCl pre-contracted tone**				
**+Endothelium**	1.93±0.40	98±1.5		
**−Endothelium**	2.02±0.23	97.1±2.2		

**NPS 2143 on - Endothelium pre-contracted vessels**				
**Methoxamine**	2.97±0.34		99.1±3.2	
**KCl**	12.5±0.73^a^		78.4±3.4^a^	

**Calindol on 10** **µM Methoxamine pre-contracted tone**				
**+Endothelium**	2.06±0.24			96.48±1.4
**−Endothelium**	2.66±0.30			94.4±2.8
**+3** **µM Calhex−231**	2.58±0.35			97.2±1.1

**Calindol on 60** **mM KCl pre-contracted tone**				
**+Endothelium**	3.26±0.24^b^			82.2±2.3^b^
**−Endothelium**	3.41±0.32^c^			83.4±1.6^c^

a P<0.05 vs. the effect of NPS 2143 on 10 µm Methoxamine pre-contracted tone.

b P<0.05 vs. the effect of calindol on 10 µm Methoxamine precontracted tone in vessels with a functional endothelium (+endothelium).

c P<0.01 vs. the effect of calindol on 10 µm Methoxamine precontracted tone in vessels lacking a functional endothelium (−endothelium).

## Appendix A Supplementary material

Supplementary material Supplementary Figure 1. Effect of Nicardipine on Methoxamine- and KCl-induced pre-contracted arteries. (A) Representative trace and (B) Bar graph of mean data showing that 1 µm nicardipine abolishes 60 mM KCl-induced pre-contracted arteries. (C) Representative trace and (D) mean data showing that nicardipine also abolishes methoxamine-induced pre-contracted arteries. n=4 animals, with at least n=3 vessel segments from each animal. ****P<0.0001.

## References

[bib1] Alam M., Kirton J.P., Wilkinson F.L., Towers E., Sinha S., Rouhi M., Vizard T.N., Sage A.P., Martin D., Ward D.T., Alexander M.Y., Riccardi D., Canfield A.E. (2009). Calcification is associated with loss of functional calcium-sensing receptor in vascular smooth muscle cells. Cardiovasc. Res..

[bib2] Atchison D.K., Ortiz-Capisano M.C., Beierwaltes W.H. (2010). Acute activation of the calcium-sensing receptor inhibits plasma renin activity in vivo. AJP Regul. Integr. Comp. Physiol..

[bib3] Awumey E.M., Bridges L.E., Williams C.L., Diz D.I. (2013). Nitric-oxide synthase knockout modulates Ca^2+^-sensing receptor expression and signaling in mouse mesenteric arteries. J. Pharmacol. Exp. Ther..

[bib4] Bonomini M., Giardinelli A., Morabito C., Di Silvestre S., Di Cesare M., Di Pietro N., Sirolli V., Formoso G., Amoroso L., Mariggiò M.A., Pandolfi A. (2012). Calcimimetic R-568 and its enantiomer S-568 increase nitric oxide release in human endothelial cells. PLoS One.

[bib5] Brown E.M., MacLeod R.J. (2001). Extracellular calcium sensing and extracellular calcium signaling. Physiol. Rev..

[bib6] Bukoski R.D., Bian K., Wang Y., Mupanomunda M. (1997). Perivascular sensory nerve Ca2+ receptor and Ca2+-induced relaxation of isolated arteries. Hypertension.

[bib7] Bukoski R.D., Bátkai S., Járai Z., Wang Y., Offertaler L., Jackson W.F., Kunos G. (2002). CB(1) receptor antagonist SR141716A inhibits Ca(2+)-induced relaxation in CB(1) receptor-deficient mice. Hypertension.

[bib8] Carrasco F.R., Pérez-Flores I., Calvo N., Ridao N., Sánchez A., Barrientos (2004). Treatment of persistent hyperparathyroidism in renal transplant patients with cinacalcet improves control of blood pressure. Transplant. Proc..

[bib9] Chen N.X., Kircelli F., O'Neill K.D., Chen X., Moe S.M. (2010). Verapamil inhibits calcification and matrix vesicle activity of bovine vascular smooth muscle cells. Kidney Int..

[bib10] Crankshaw D.J., Pistilli M.J., O'Brien Y.M., Sweeney E.M., Dockery P., Holloway A.C., Morrison J.J. (2013). The effects of extracellular calcium-sensing receptor ligands on the contractility of pregnant human myometrium in vitro. Reprod. Sci..

[bib11] Dora K.A., Gallagher N.T., McNeish A., Garland C.J. (2008). Modulation of endothelial cell KCa3.1 channels during endothelium-derived hyperpolarizing factor signaling in mesenteric resistance arteries. Circ. Res..

[bib12] Faure H., Gorojankina T., Rice N., Dauban P., Dodd R.H., Bräuner-Osborne H., Rognan D., Ruat M. (2009). Molecular determinants of non-competitive antagonist binding to the mouse GPRC6A receptor. Cell Calcium.

[bib13] Fitzpatrick L.A., Smith P.L., McBride T.A., Fries M.A., Hossain M., Dabrowski C.E., Gordon D.N. (2011). Ronacaleret, a calcium-sensing receptor antagonist, has no significant effect on radial fracture healing time: results of a randomized, double blinded, placebo-controlled Phase II clinical trial. Bone.

[bib14] Greenberg H.Z.E., Shi J., Jahan K.S., Martinucci M.C., Gilbert S.J., Vanessa Ho W.-S., Albert A.P. (2016). Stimulation of calcium-sensing receptors induces endotheliumdependent vasorelaxations via nitric oxide production and activation of IKCa channels. Vasc. Pharmacol..

[bib15] Han S.-L., Wan S.-L. (2012). Effect of teriparatide on bone mineral density and fracture in postmenopausal osteoporosis: meta-analysis of randomised controlled trials. Int. J. Clin. Pract..

[bib16] Hebert S.C. (2006). Therapeutic use of calcimimetics. Annu. Rev. Med..

[bib17] Hofer A.M., Brown E.M. (2003). Extracellular calcium sensing and signalling. Nat. Rev. Mol. Cell Biol..

[bib18] Ishioka N., Bukoski R.D. (1999). A role for N-arachidonylethanolamine (anandamide) as the mediator of sensory nerve-dependent Ca2+-induced relaxation. J. Pharmacol. Exp. Ther..

[bib19] Jensen A.A., Bräuner-Osborne H. (2007). Allosteric modulation of the calcium-sensing receptor. Curr. Neuropharmacol..

[bib20] Johansson H., Cailly T., Rojas Bie Thomsen A., Bräuner-Osborne H., Sejer Pedersen D. (2013). Synthesis of the calcilytic ligand NPS 2143. Beilstein J. Org. Chem..

[bib21] John M.R., Harfst E., Loeffler J., Belleli R., Mason J., Bruin G.J.M., Seuwen K., Klickstein L.B., Mindeholm L., Widler L., Kneissel M. (2014). AXT914 a novel, orallyactive parathyroid hormone-releasing drug in two early studies of healthy volunteers and postmenopausal women. Bone.

[bib22] Letz S., Haag C., Schulze E., Frank-Raue K., Raue F., Hofner B., Mayr B., Schöfl C. (2014). Amino alcohol- (NPS-2143) and quinazolinone-derived calcilytics (ATF936 and AXT914) differentially mitigate excessive signalling of calcium-sensing receptor mutants causing Bartter syndrome Type 5 and autosomal dominant hypocalcemia. PLoS One.

[bib23] Li G., Wang Q., Hao J., Xing W., Guo J., Li H., Bai S., Li H., Zhang W., Yang B., Yang G., Wu L., Wang R., Xu C. (2011). The functional expression of extracellular calcium-sensing receptor in rat pulmonary artery smooth muscle cells. J. Biomed. Sci..

[bib24] Loot A.E., Pierson I., Syzonenko T., Elgheznawy A., Randriamboavonjy V., Zivković A., Stark H., Fleming I. (2013). Ca2+-sensing receptor cleavage by calpain partially accounts for altered vascular reactivity in mice fed a high-fat diet. J. Cardiovasc. Pharmacol..

[bib25] Mancilla E.E., De Luca F., B.J. (1998). Activating mutations of the Ca2+-sensing receptor. Mol. Genet. Metab..

[bib26] Molostvov G., James S., Fletcher S., Bennett J., Lehnert H., Bland R., Zehnder D. (2007). Extracellular calcium-sensing receptor is functionally expressed in human artery. Am. J. Physiol. Ren. Physiol..

[bib27] Molostvov G., Fletcher S., Bland R., Zehnder D. (2008). Extracellular calcium-sensing receptor mediated signalling is involved in human vascular smooth muscle cell proliferation and apoptosis. Cell. Physiol. Biochem..

[bib28] Mulvany M.J., Halpern W. (1977). Contractile properties of small arterial resistance vessels in spontaneously hypertensive and normotensive rats. Circ. Res..

[bib29] Mupanomunda M.M., Wang Y., Bukoski R.D. (1998). Effect of chronic sensory denervation on Ca(2+)-induced relaxation of isolated mesenteric resistance arteries. Am. J. Physiol..

[bib30] Nemeth E.F., Steffey M.E., Hammerland L.G., Hung B.C., Van Wagenen B.C., DelMar E.G., Balandrin M.F. (1998). Calcimimetics with potent and selective activity on the parathyroid calcium receptor. Proc. Natl. Acad. Sci. USA.

[bib31] Nemeth E.F., Delmar E.G., Heaton W.L., Miller M.A., Lambert L.D., Conklin R.L., Gowen M., Gleason J.G., Bhatnagar P.K., Fox J. (2001). Calcilytic compounds: potent and selective Ca2+ receptor antagonists that stimulate secretion of parathyroid hormone. J. Pharmacol. Exp. Ther..

[bib32] Odenwald T., Nakagawa K., Hadtstein C., Roesch F., Gohlke P., Ritz E., Schaefer F., Schmitt C.P. (2006). Acute blood pressure effects and chronic hypotensive action of calcimimetics in uremic rats. J. Am. Soc. Nephrol..

[bib33] Ogata H., Ritz E., Odoni G., Amann K., Orth S.R. (2003). Beneficial effects of calcimimetics on progression of renal failure and cardiovascular risk factors. J. Am. Soc. Nephrol..

[bib34] Petrel C., Kessler A., Maslah F., Dauban P., Dodd R.H., Rognan D., Ruat M. (2003). Modeling and mutagenesis of the binding site of Calhex 231, a novel negative allosteric modulator of the extracellular Ca(2+)-sensing receptor. J. Biol. Chem..

[bib35] Petrel C., Kessler A., Dauban P., Dodd R.H., Rognan D., Ruat M. (2004). Positive and negative allosteric modulators of the Ca2+-sensing receptor interact within overlapping but not identical binding sites in the transmembrane domain. J. Biol. Chem..

[bib36] Rich S., Kaufmann E., Levy P.S. (1992). The effect of high doses of calcium-channel blockers on survival in primary pulmonary hypertension. N. Engl. J. Med..

[bib37] Rybczynska A., Boblewski K., Lehmann A., Orlewska C., Foks H., Drewnowska K., Hoppe A. (2005). Calcimimetic NPS R-568 induces hypotensive effect in spontaneously hypertensive rats. Am. J. Hypertens..

[bib38] Rybczyńska A., Boblewski K., Lehmann A., Orlewska C., Foks H. (2006). Pharmacological activity of calcimimetic NPS R-568 administered intravenously in rats: dose dependency. Pharmacol. Rep..

[bib39] Rybczynska A., Lehmann A., Jurska-Jasko A., Boblewski K., Orlewska C., Foks H., Drewnowska K. (2006). Hypertensive effect of calcilytic NPS 2143 administration in rats. J. Endocrinol..

[bib40] Rybczynska A., Jurska-Jasko A., Boblewski K., Lehmann A., Orlewska C. (2010). Blockade of calcium channels and AT1 receptor prevents the hypertensive effect of calcilytic NPS 2143 in rats. J. Physiol. Pharm..

[bib41] Saidak Z., Brazier M., Kamel S., Mentaverri R. (2009). Agonists and allosteric modulators of the calcium-sensing receptor and their therapeutic applications. Mol. Pharmacol..

[bib42] Schepelmann M., Yarova P.L., Lopez-Fernandez I., Davies T.S., Brennan S.C., Edwards P.J., Aggarwal A., Graça J., Rietdorf K., Matchkov V., Fenton R.A., Chang W., Krssak M., Stewart A., Broadley K.J., Ward D.T., Price S.A., Edwards D.H., Kemp P.J., Riccardi D. (2016). The vascular Ca2+-sensing receptor regulates blood vessel tone and blood pressure. Am. J. Physiol. Cell Physiol..

[bib43] Sitbon O., Humbert M., Jaïs X., Ioos V., Hamid A.M., Provencher S., Garcia G., Parent F., Hervé P., Simonneau G. (2005). Long-term response to calcium channel blockers in idiopathic pulmonary arterial hypertension. Circulation.

[bib44] Smajilovic S., Hansen J.L., Christoffersen T.E.H., Lewin E., Sheikh S.P., Terwilliger E.F., Brown E.M., Haunso S., Tfelt-Hansen J. (2006). Extracellular calcium sensing in rat aortic vascular smooth muscle cells. Biochem. Biophys. Res. Commun..

[bib45] Smajilovic S., Sheykhzade M., Holmegard H.N., Haunso S., Tfelt-Hansen J. (2007). Calcimimetic, AMG 073, induces relaxation on isolated rat aorta. Vasc. Pharmacol..

[bib46] Smajilovic S., Yano S., Jabbari R., Tfelt-Hansen J. (2011). The calcium-sensing receptor and calcimimetics in blood pressure modulation. Br. J. Pharm..

[bib47] Steddon C.J., Cunningham D.M. (2005). Calcimimetics and calcilytics--fooling the calcium receptor. Lancet.

[bib48] Thakore P., Ho W.-S.V. (2011). Vascular actions of calcimimetics: role of Ca^2^(+) -sensing receptors versus Ca^2^(+) influx through lLs. Br. J. Pharm..

[bib49] Wang Y., Bukoski R.D. (1998). Distribution of the perivascular nerve Ca2+ receptor in rat arteries. Br. J. Pharm..

[bib50] Ward D.T., Riccardi D. (2012). New concepts in calcium-sensing receptor pharmacology and signalling. Br. J. Pharm..

[bib51] Weston A., Absi M., Ward D., Ohanian J., Dodd R., Dauban P., Petrel C., Ruat M., Edwards G. (2005). Evidence in favor of a calcium-sensing receptor in arterial endothelial cells: studies with calindol and Calhex 231. Circ. Res..

[bib52] Weston A.H., Absi M., Harno E., Geraghty A.R., Ward D.T., Ruat M., Dodd R.H., Dauban P., Edwards G. (2008). The expression and function of Ca(2+)-sensing receptors in rat mesenteric artery; comparative studies using a model of type II diabetes. Br. J. Pharm..

[bib53] Wonneberger K., Scofield M.A., Wangemann P. (2000). Evidence for a calcium-sensing receptor in the vascular smooth muscle cells of the spiral modiolar artery. J. Membr. Biol..

[bib54] Yamamura A., Guo Q., Yamamura H., Zimnicka A.M., Pohl N.M., Smith K.A., Fernandez R.A., Zeifman A., Makino A., Dong H., Yuan J.X.-J. (2012). Enhanced Ca(2+)-sensing receptor function in idiopathic pulmonary arterial hypertension. Circ. Res..

[bib55] Yamamura A., Yamamura H., Guo Q., Zimnicka A.M., Wan J., Ko E.A., Smith K.A., Pohl N.M., Song S., Zeifman A., Makino A., Yuan J.X.-J. (2013). Dihydropyridine Ca(2+) channel blockers increase cytosolic [Ca(2+)] by activating Ca(2+)-sensing receptors in pulmonary arterial smooth muscle cells. Circ. Res..

[bib56] Yamamura A., Ohara N., Tsukamoto K. (2015). Inhibition of excessive cell proliferation by calcilytics in idiopathic pulmonary arterial hypertension. PLoS One.

[bib57] Yamamura A., Yagi S., Ohara N., Tsukamoto K. (2016). Calcilytics enhance sildenafilinduced antiproliferation in idiopathic pulmonary arterial hypertension. Eur. J. Pharmacol..

[bib58] Yarova P.L., Stewart A.L., Sathish V., Britt R.D., Thompson M.A., P Lowe A.P., Freeman M., Aravamudan B., Kita H., Brennan S.C., Schepelmann M., Davies T., Yung S., Cholisoh Z., Kidd E.J., Ford W.R., Broadley K.J., Rietdorf K., Chang W., Bin Khayat M.E., Ward D.T., Corrigan C.J., T Ward J.P., Kemp P.J., Pabelick C.M., Prakash Y.S., Riccardi D. (2015). Calcium-sensing receptor antagonists abrogate airway hyperresponsiveness and inflammation in allergic asthma. Sci. Transl. Med..

[bib59] Ziegelstein R.C., Xiong Y., He C., Hu Q. (2006). Expression of a functional extracellular calcium-sensing receptor in human aortic endothelial cells. Biochem. Biophys. Res. Commun..

