# Supplementary figures and images for: The calcilytics Calhex-231 and NPS 2143 and the calcimimetic Calindol reduce vascular reactivity via inhibition of voltage-gated Ca2+ channels
Source: Eur J Pharmacol. 2016 Nov 15;791:659–68. doi: 10.1016/j.ejphar.2016.10.008 (PMC5127511; doi:10.1016/j.ejphar.2016.10.008)

# Supplementary Figure 1

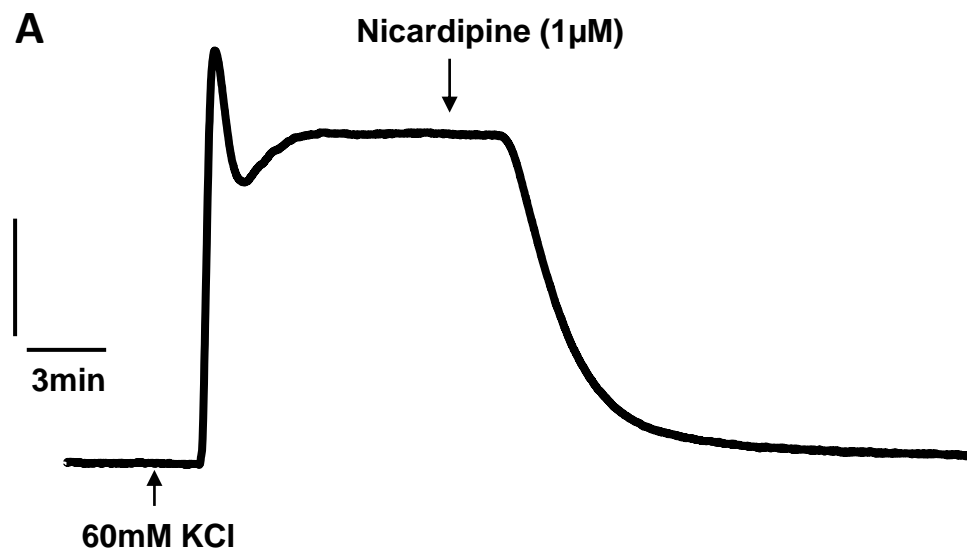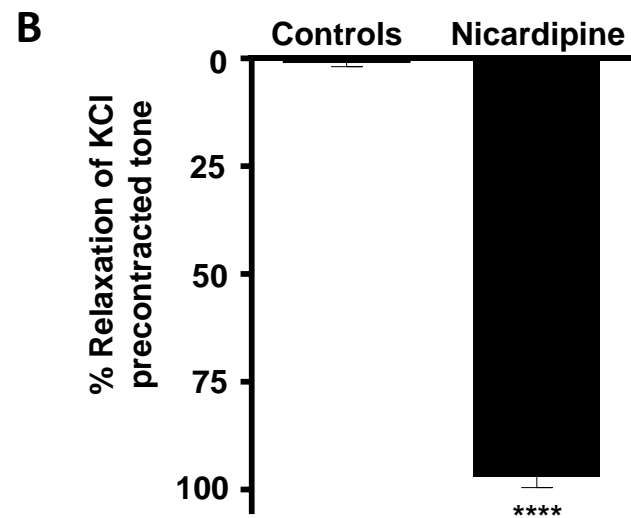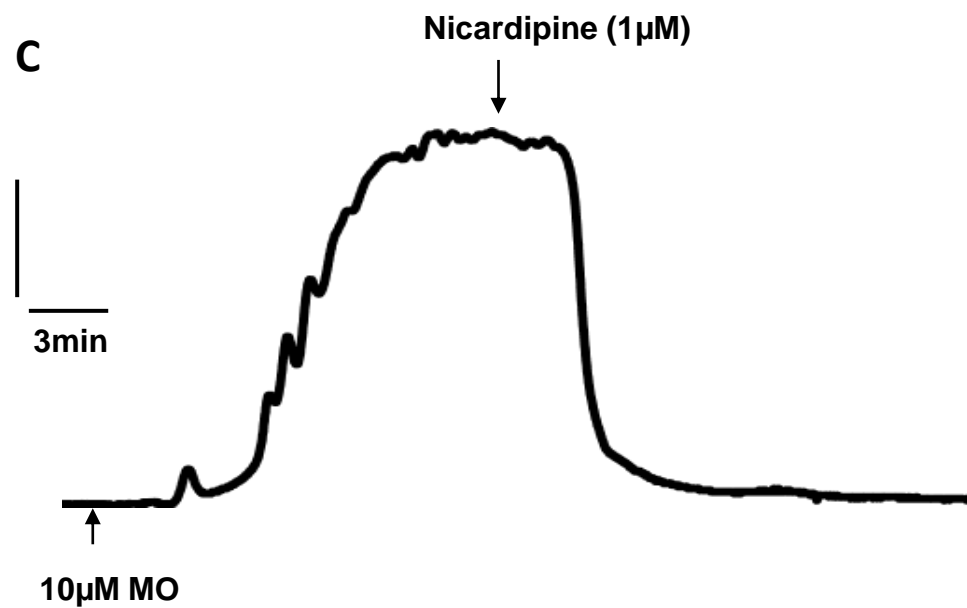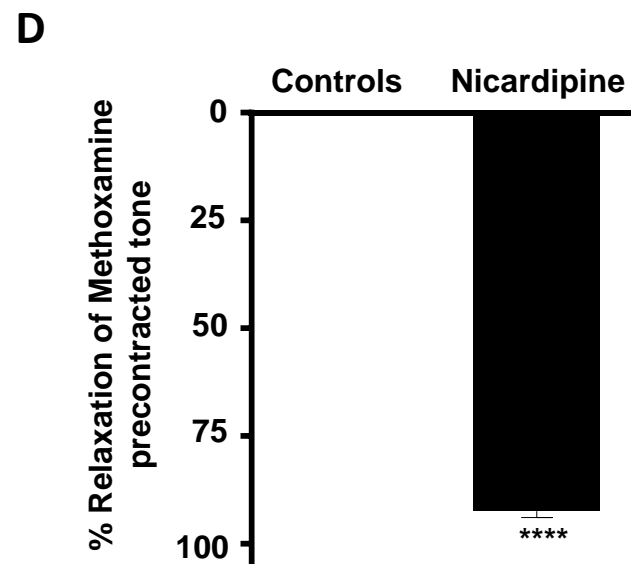

Supplement: Supplementary file 1 — Supplementary material Supplementary Figure 1. Effect of Nicardipine on Methoxamine- and KCl-induced pre-contracted arteries. (A) Representative trace and (B) Bar graph of mean data showing that 1 µm nicardipine abolishes 60 mM KCl-induced pre-contracted arteries. (C) Representative trace and (D) mean data showing that nicardipine also abolishes methoxamine-induced pre-contracted arteries. n=4 animals, with at least n=3 vessel segments from each animal. ****P<0.0001. [file mmc1.pdf]
